# 

**Published:** 1891-09-26

**Authors:** 


### NOTICE TO CORRESPONDENTS.

All MS., Letters, Books for Review, and other matters intended for the Editor should be addressed The Editor, The Lodge, Porchester Square, London, W.

The Editor cannot undertake to return rejected M.S., even when accompanied by stamped directed envelope.

**Notice to Advertisers and Subscribers.**

All Advertisements, Orders for Copies of The Hospital, and Business Communications should be addressed to The Publisher, *not to the Editor*, The Hospital, 140, Strand, W.C.

The Cost of Subscription, post free, is as follows:—Per week, 1½d.; per quarter, 1s. 8d.; per half-year, 3s. 3d.; per annum, 6s. 6d. Foreign:—Per annum, 8s. 8d.; payable, in all cases, in advance.

**NOTICE.**—The Index for Vol. IX., (ending March, 1891) is on sale at the Office of this Journal, price 2½d., post free.

### GENERAL CHARITIES.

[This Column is devoted to an account of work of charitable institutions other than Medical Charities. The Editor will be glad to receive reports or news of work at 140, Strand. *Philanthropy* should be plainly written on the envelope.]

Some industrial schools now form part of the establishment of the London School Board. Among them is **THE BRENTWOOD INDUSTRIAL SCHOOL**, which is directed to the reclamation of boys, chargeable to London, in danger of falling into evil courses. The latest report of the Committee, of whom Miss Davenport Hill is an active member, is highly creditable to the boys. Since 1874 there have been 280 boys discharged, all of whom had been committed under magistrates' orders, and all are known to be doing well. This is certainly a remarkable record, which, according to a report just sent us by the Deputy-Inspector as to the behaviour of the boys now in the school, is in no danger of being upset. He writes in the highest terms of the general work of the school, and of the health and happiness of the boys. The drainage has just been renewed, and the building thoroughly repaired. The boys make their own clothes, boots, and bread, and have an excellent band. It appears that this band is a large feeder of the army, as fully 50 per cent. join it; and the reports from the various regiments who have received recruits for their bands from this quarter have been most satisfactory. The school is not a penal asylum, but every effort is made that the boys shall feel it is a home from which they can start in life. Some are as young as eight years of age when they first enter the school, which opens its doors also to the halt and the maimed, who are successfully educated, and fitted for an industrial life.

The work embraced in the circle of operations of **THE CHILDREN'S HOME** is wide and varied. One peculiar feature, peculiar because it is not common, in the work, is the employment of women in the domestic management and moral training of the boys. Dr. Stephenson has adopted this plan on the ground that the best influences of a boy's early life come from his mother. He protests against the conception that any decent woman will do for such work as this. Not his experience only, but that of all who have had much to do with the training of the young, proves that for the moral training of children, especially of children whose minds have been warped, either through neglect or through evil influences, there is needed a careful and delicate and discriminating touch. Though there may not be so many children to be found actually sleeping in the open air, or living a neglected life, as was the case a generation ago, it would be a questionable affirmation that the lives of children in our large towns are not surrounded still by as many dangerous circumstances as ever. The nature of the circumstances may and no doubt does, differ from time to time, but we fear that so long as the world exists, the miseries we daily witness will never be eradicated, though they may be diminished by agencies for the rescue of children, such as this and other kindred institutions. The Children's Home has branches at Gravesend, Isle of Man, Birmingham, and in Canada, and the London home is in Bonner Road, Victoria Park, E. In this branch there were 305 children in residence at the close of last year, and of the children sent out 54 have been transferred to other branches, 44 have been emigrated to Canada, and 28 have been provided for in England. The work in the various industrial departments is found to have the best influence on the habits and conduct of the children.

The latest report of the **FELTHAM INDUSTRIAL SCHOOL** is a favourable one. According to the Inspector it is well organised and administered in every detail, and regulated with caution and prudence. We venture, however, to think that the policy of diminishing the amount of school-room space when the number of inmates is much in excess of any total that has been in the place for many years is a very questionable one. The existing school-rooms are, as a result, liable to be overcrowded, which is detrimental to efficiency and the requisite training, not to mention the fact of the unhealthiness of overcrowding a lot of boys, which is likely to be attended with an increased amount of sickness. The Medical Officer of Health in his last report pointed out that diseases of the respiratory organs occupy too large a space in the list of disease. No doubt the management will carry out, if they have not already done so, the suggestion of the Medical Officer that new clothing should be issued, and the chest coverings of the farm boys be increased. The school is now divided into four sections of 180 boys, with a depot for the reception of new boys in the West House. The construction of two new dining halls, each capable of accommodating 360 boys, has added greatly to their comfort, as they now can have their meals served hot in well-warmed and ventilated rooms, without the inconvenience from overcrowding. The discipline of the boys seems to have been good, with two exceptions, when two batches absconded; but not, it appears, on account of any improper treatment experienced by the boys under detention. It is the opinion of the Superintendent that the school has suffered materially, so far as discipline is concerned, by the licensing of the older and more responsible boys. A feature of the school is the farm, which during the past year yielded a profit of £150. In addition to farming, the boys learn various trades, are taught swimming, and have a gymnasium. The average cost per boy for maintenance, including food, clothing, and management, amounts to about £23 5s.

### SCRAPS AND GLEANINGS.

GRAYS intends to have a cottage hospital.

DR. ALICE MACLAREN and several of the nurses at Leith are down with scarlet fever.

INFLUENZA is very prevalent in Spain, at Kaen 5,000 persons are said to be down with it.

AFTER some hot discussion Mr. Wilks's appointment to Salisbury Infirmary has been confirmed.

CONAN DOYLE, novelist and doctor, has been writing in the *Daily Telegraph* in favour of total prohibition.

A PORTRAIT of the Countess of Shaftesbury was unveiled in the Belfast Royal Hospital, on September 11th.

DENIS, an attendant at Broadmoor, has been almost murdered by a patient. Luckily another patient came to the rescue.

THE Meridan Workhouse Infirmary for twelve beds was opened lately in good old English fashion with a treat to the inmates.

THE half-yearly meeting of the Governors of the Wolverhampton Hospital was held on September 8th, when some of the rules were slightly altered.

AT a meeting held in Portsmouth last week, it was decided to found an annual festival in aid of the Portsmouth hospitals and other charitable institutions.

STRICTURES on the hospital management of the Liskeard Board of Guardians have been passed by the Inspector. The wards were dirty and the nursing most faulty.

PROFESSOR VIRCHOW has been examining the skulls of the ancient Greeks, and finds them small; evidently though the most intellectual race, they had not the largest quantity of brains.

IT is intended to ornament the interior of the new Cottage Hospital, Ormesby, with the assistance of the men of the various works. The decorations are to be inscribed with the names of the district works contributing.

THE return of metropolitan pauperism for the second week of August shows a continued decrease as compared with the three previous years. The total number of paupers was 85,092, including 53,962 indoor and 31,130 outdoor.

AN old lady, named Margaret M'Gunnall, has died in Eastry Workhouse at the age of 102 years, having been born in 1779. She was a native of Ross, in Scotland, was the wife of an old coastguardsman, and had been in Eastry Workhouse thirty-seven years.

THE discussion on trained versus pauper nurses for workhouses which lately took place at Gilsland would seem to be bearing good fruit. The Whitehaven Guardians have decided to employ trained nurses, and the subject is soon to be brought before the Wigtown Guardians.

THE *Daily Telegraph* says that there is not the slightest foundation for the report that cholera had broken out with increased violence at El Tor, and that numerous victims had been carried off during the past fortnight, especially among the pilgrims returning from Mecca.

DR. MURDOCH CAMERON has operated upon his thirteenth case of Cæsarean section. The patient was rachitic, and was sent from a neighbouring town, after being three days in labour. Both mother and child are doing well. The latter weighed eight pound, and is at the breast.

THE Committee of the Seamen's Hospital Society have decided to open a new dispensary for sailors in East India Dock Road, on October 1st, to meet the needs of the largely-increasing number of seamen who are now congregating in the neighbourhood. The Society appeals for additional funds.

THE little Hospital of St. Saviour's, Osnaburgh Street, Regent's Park, is quite unique in its treatment of cancer and other complaints to which women are specially liable. No knife operation is used even in the severest cases; nevertheless, it is stated a cure is effected in the majority of cases.

AN outbreak of smallpox occurred last week on board her Majesty's ship "Collingwood," at Thaso. All communication with the shore was immediately stopped and the ship ordered to proceed to Salonica, where there is a hospital for infectious diseases. It is believed that the disease was contracted at Alexandria.

THE Friendly Societies of the North-East show their independence in a strange fashion. They ask for grants from the Auckland Board of Guardians, but object to issuing tickets in return for a subscription, as is the custom in the case of other institutions, considering such a measure as likely to pauperise the Home.

DOCTOR GRECHEN, of Luxembourg, who was recently fined 5,000 marks for publishing a pamphlet describing the diseases of distinguished patients, and giving their full initials, has issued a second edition of the scandalous pamphlet in which the initials are simply reversed. The excitement caused by the publication is great. The doctor's life is threatened.

THE eighth annual report of the Haydock Cottage Hospital shows that the institution has continued to do good work, and is looked upon with increasing favour. The new mortuary is almost completed, and the funds for the complete equipment of the Hospital with surgical implements and apparatus are nearly provided, a further sum of £30 only being now needed.

A DISTRIBUTION of awards to persons who distinguished themselves in saving life in connection with the *Utopia* disaster has taken place at Gibraltar. Mr. Cavendish Boyle, C.M.G., received a testimonial on vellum from the Royal Humane Society for having by his excellent arrangements helped to save many lives. Mr. Seed, Chief of Police, received a silver medal for similar services, and he, with Corporal M'Gee, also received the Albert Medal. Two boarding officers and six men of the Port Department were presented with medals by the Board of Trade and the Royal Humane Society.

### THE STUDENT OF MEDICINE.

[All communications for this Supplement should be addressed to The Editor of The Hospital, at 140, Strand, with the words, "Students' Column," written in the left hand top corner of the envelope.]

### APPOINTMENTS.

At the Royal Infirmary, Manchester, Albert Edward Berry, M.B. Lond., M.R.C.S. Eng., L.R.C.P. Lond., has been appointed house surgeon. At the General Infirmary, Leeds, H. E. Tomlinson has been appointed house physician. At the Royal United Hospital, Bath, Leslie A. Walsh, M.R.C.S., L.R.C.P., L.S.A., has been appointed house surgeon.

### VACANCIES.

The following vacancies are announced this week, the amount of salary in each case being given after the name of the institution:

### Resident Medical Officers.

Bethlem Hospital, S.E., Two Resident Clinical Assistants—Board.

Bolton Infirmary and Dispensary, Junior House Surgeon, Salary £100 per year, rising £10 annually to £150, with furnished apartments and board, &c.

Burton-on-Trent Infirmary, House Surgeon—Salary £130 per annum, with prospect of increase.

Cheshire County Asylum, Upton, near Chester, Junior Assistant Medical Officer—Salary £120 and board.

City of London Hospital for Diseases of the Chest, Victoria Park, E., House Physician—Board, &c.

Eastern Counties Asylum for Idiots, Colchester, Resident Medical Attendant- Salary £100 and board.

Hull Royal Infirmary, House Surgeon—Salary £100 and board.

Royal Free Hospital, Gray's Inn Road, Junior Resident Medical Officer—Board, &c.

### Non-Resident Medical Officers.

Charing Cross Hospital, Strand, W.C., Surgical Registrar—Salary £40.

Dental Hospital of London, Leicester Square, Anæsthetist and Assistant Anæsthetist.

King's College, London, Demonstrator of Bacteriology.

Middlesex Hospital, Pathologist and Curator of the Museum.

Western Ophthalmic Hospital, 153, Marylebone Road, W., Assistant Surgeon and Assistant Surgeon to act as Clinical Assistant to the Surgeons.

Westminster Hospital, Broad Sanctuary, S.W., Second Dental Surgeon.

### PASS LISTS.

### University of Durham.

The following candidates at the First Examination for the Degree of Bachelor in Medicine at the College of Medicine, Newcastle-on-Tyne, have satisfied the Examiners:—

Elementary Anatomy and Physiology, Chemistry, and Botany. —W. E. Alderson, Norman Bennett, Wm. A. I'Anson Charlton, Percival Davidson, Leopold Fothergill, Gilbert Gocher, Wm. T. Harkness, Thos. Horton, Harald Guthrie McAllum, William Wilkie Ridley, Tom Sanderson, Geo. William Scott, Leonard King Tickner, Percy Hugh Wardell-Johnson, all of the College of Medicine, Newcastle-on-Tyne.

Elementary Anatomy and Physiology.—Richard A. Mills-Roberts, College of Medicine, Newcastle-on-Tyne; Walter Geo. Parkinson, Yorkshire College, Leeds; Joseph Parker Woodhouse, University College, Liverpool.

Chemistry and Botany.—Chas. Harold Godwin Atkyns, Jas. Eder Harper, Chas. Holmes Joy, Frank Laughton-Smith, Frank Morris, E. P. Satchell, and G. Headly Tomlinson, all of Queen's College, Birmingham; Harry Bond, Harry Crichton, Geo. Wm. Harbottle, Alfred P. Lloyd, E. Tonge, T. Huddlestone Urwin, and W. Mott Whitehouse, all of the College of Medicine, Newcastle-on-Tyne; C. A. Brough, Edinburgh School of Medicine; Wm. Thos. Beddoes, Donnelly, Gerardo Jimenez, Fred William Rowland, Walter John Rowland, and Federico Zumbado, all of Guy's Hospital; H. Stagg Byers, E. W. Everett, Percy Lambert, and R. C. Jeremie Stevens, all of St. Bartholomew's Hospital; Alfred James Grant and Harold Huskinson, both of St. Thomas's Hospital; Edw. W. Joscelyne, St. Mary's Hospital; Alfred Cornelius Leigh, University College, London; Bertie James Mayne, Middlesex Hospital; John Ralph Prior, King's College Hospital.

Chemistry.—George Elam, M.R.C.S. Eng., L.R.C.P. Lond., and Wm. Ernest Jones, M.R.C.S. Eng., L.R.C P. Lond., of Middlesex Hospital; Henry John Mathews, L.R.C.S., L.R C.P. Irel., Royal College of Surgeons, Ireland; Edward Fredk. Pratt., L.R.C.P. Lond., Queen's College, Birmingham.

### Indian Medical Service.

The following is a list of the candidates for her Majesty's Indian Medical Service who were successful at the competitive examination held at Burlington House on August 24th:—

| | Marks. | | Marks. |
|---|---|---|---|
| Seton, B. G. ... ... | 3,650 | Sprague, W. C. ... ... | 3 200 |
| Armstrong, W. E. A. ... | 3,264 | Mitter, R. K. ... ... | 3,155 |
| Elliot, R. H. ... ... | 3,240 | Yeates, R. A. ... ... | 3,105 |

Twenty-six candidates competed for six appointments. All were reported qualified.

